# Regulation and Functional Complexity of the Chlorophyll-Binding Protein IsiA

**DOI:** 10.3389/fmicb.2021.774107

**Published:** 2021-11-17

**Authors:** Anqi Jia, Yanli Zheng, Hui Chen, Qiang Wang

**Affiliations:** State Key Laboratory of Crop Stress Adaptation and Improvement, School of Life Sciences, Henan University, Kaifeng, China

**Keywords:** CP43, cyanobacteria, iron deficiency, IsiA, photosynthesis

## Abstract

As the oldest known lineage of oxygen-releasing photosynthetic organisms, cyanobacteria play the key roles in helping shaping the ecology of Earth. Iron is an ideal transition metal for redox reactions in biological systems. Cyanobacteria frequently encounter iron deficiency due to the environmental oxidation of ferrous ions to ferric ions, which are highly insoluble at physiological pH. A series of responses, including architectural changes to the photosynthetic membranes, allow cyanobacteria to withstand this condition and maintain photosynthesis. Iron-stress-induced protein A (IsiA) is homologous to the cyanobacterial chlorophyll (Chl)-binding protein, photosystem II core antenna protein CP43. IsiA is the major Chl-containing protein in iron-starved cyanobacteria, binding up to 50% of the Chl in these cells, and this Chl can be released from IsiA for the reconstruction of photosystems during the recovery from iron limitation. The pigment–protein complex (CPVI-4) encoded by *isiA* was identified and found to be expressed under iron-deficient conditions nearly 30years ago. However, its precise function is unknown, partially due to its complex regulation; *isiA* expression is induced by various types of stresses and abnormal physiological states besides iron deficiency. Furthermore, IsiA forms a range of complexes that perform different functions. In this article, we describe progress in understanding the regulation and functions of IsiA based on laboratory research using model cyanobacteria.

## Introduction

Cyanobacteria are the oldest known lineage of oxygen-releasing photosynthetic organisms (Fournier et al., 2021). Oxygenic photosynthesis, which was first invented by cyanobacteria, is widely thought to have led to the ancient conversion of the reducing atmosphere into an oxidizing one (Nealson and Myers, 1990; Wang et al., 2017; Catling and Zahnle, 2020). Then, oxidative phosphorylation provides energy support for the evolution of larger individuals and the formation of the ozone layer made it possible for life to come ashore (Vik, 2007; Jie et al., 2019). Cyanobacteria are evolutionary ancestors to chloroplasts, and the comparison of chloroplasts and cyanobacteria showed their similarities (Tomitani, 1999). Thus, cyanobacteria helped shaping the ecology of this blue planet more than any other group of organisms until humans developed modern technologies.

Fe, the fourth most common elements in the earth’s crust, mostly exists in the form of ferrous iron. Iron can have multiple, coordination-dependent electrical potentials (−2 to +6), making it an ideal transition metal for redox reactions in biological systems (Guerinot and Yi, 1994). Iron involved in basic biochemical functions in early life, including nitrogen reduction, pigment synthesis and degradation, fatty acid metabolism, and DNA synthesis, which created an irreversible link between Fe and life (Wrigglesworth and Baum, 1980). Accordingly, cyanobacteria have heavily incorporated iron into their basic electron transport systems for photosynthesis. The photosynthetic apparatus therefore represents one of the most iron-enriched cellular systems, requiring 23 to 24 atoms of iron in a single linear electron transport chain, including 12 iron atoms in the 4Fe-4S centers of PSI (Ferreira and Straus, 1994; Raven et al., 1999; Behrenfeld and Milligan, 2013; Guowei et al., 2021).

During oxidization, ferrous iron is oxidized to ferric iron, which is hardly soluble in water and has low bioavailability at a physiological pH (Guerinot and Yi, 1994). Iron deficiency is therefore a common stress encountered by cyanobacteria, which can lead to a significant decrease in their chlorophyll (Chl)-binding protein contents (Guikema and Sherman, 1984). Cyanobacteria have evolved several responses that allow them to withstand this physiological crisis and maintain photosynthesis under iron deficiency. In response to limited iron bioavailability, the phycocyanin (PC) and Chl a contents of cyanobacteria are reduced, along with a large decrease in the number of phycobilisomes, the main light harvesting proteins of PSII embedded in the thylakoid membrane (Guikema and Sherman, 1983; Sherman and Sherman, 1983; Guikema and Sherman, 1984; Pakrasi et al., 1985a; Odom et al., 1993; Falk et al., 1995). As the number of iron-containing proteins, such as Fd and Cytc553, decreases, the iron-stress-induced proteins, such as flavodoxin, PC, IsiA, IdiA (iron deficiency induced protein A), and OCP (orange carotenoid protein), become more abundant (Laudenbach and Straus, 1988; Odom et al., 1993; Exss-Sonne et al., 2000; Sandström et al., 2002; Wilson et al., 2006). Iron deficiency also results in carotenoid (Car) accumulation and enhances fatty acid desaturation (Ivanov et al., 2007). Along with these diverse structural and compositional changes, iron deficiency affects many photochemical properties of PSII and PSI, resulting in a decrease in the PSI/PSII ratio. This decrease inhibits linear intersystem electron transport but enhances cyclic electron transport around PSI (Ivanov et al., 2000; Sandström et al., 2002) and reduces the capacity for state transitions, locking the cyanobacteria in state I, in which PSI absorbs more light energy than PSII, and more excitation energy is transferred to PSII (Ivanov et al., 2006).

Among the proteins involved in the response to limited iron bioavailability, IsiA is an excellent marker of iron deficiency, as it is strongly induced and becomes the most abundant Chl-binding protein in iron-starved cells (Burnap et al., 1993). Over the past 30years, extensive research has been performed on IsiA, but its exact function has not been determined. Table 1 lists the nine most highly co-cited articles and the main findings of these articles. From these top-cited publications, we clearly observed the evolution of IsiA research over time. The *isiA* gene was first reported by Laudenbach and Straus in 1988 (Laudenbach and Straus, 1988), with the protein product identified in 1993 (Burnap et al., 1993). IsiA was initially proposed to protect PSII from excessive excitation (Park et al., 1999; Sandström et al., 2001); however, two groups simultaneously identified the IsiA_18_-PSI trimer complex in two model cyanobacteria species (Bibby et al., 2001a; Boekema et al., 2001), with later studies demonstrating that IsiA was efficiently coupled to the PSI reaction center core (Andrizhiyevskaya et al., 2002; Melkozernov et al., 2003). Subsequently, it was discovered that IsiA formed various complexes and had dual functions as an energy collector and an energy dissipater under prolonged iron deficiency (Yeremenko et al., 2004). These results represent the intellectual base of IsiA research.

**Table 1:** The nine most highly co-cited references published before 2021.

Rank	Number of co-citations	References	Main results
1	275	Bibby et al. (2001a) *Nature*, 412, 743–745	Iron deficiency induces the formation of IsiA_18_-PSI_3_ in *Synechocystis* sp. PCC 6803
2	260	Boekema et al. (2001) *Nature*, 412, 745–748	Iron deficiency induces the formation of IsiA_18_–PSI_3_ in *Synechococcus* sp. PCC 7942
3	107	Yeremenko et al. (2004) *Biochemistry*, 43, 10,308–10,313	IsiA forms various complexes and functions as an energy collector and dissipater during prolonged iron stress
4	99	Park et al. (1999) *Molecular Microbiology*, 32, 123–129	IsiA protects photosystem II from excess light under iron-limited conditions
5	95	Havaux et al. (2005) *FEBS Letters*, 579, 2,289–2,292	IsiA protects cyanobacteria from photooxidative stress and plays a photoprotective role
6	74	Melkozernovet al. (2003) *Biochemistry*, 42, 3,893–3,903	The IsiA antenna ring is efficiently coupled to the PSI reaction center core
7	73	Andrizhiyevskaya et al. (2002) *BBA-Bioenergetics*, 1,556, 265–272	The IsiA ring increases the absorption cross section of PSI by about 100%
8	69	Sandström et al. (2001) *Photochemistry and Photobiology*, 74, 431–437	IsiA functions as an excitation energy dissipator that protects photosystem II from excess light under iron-limited conditions
9	52	Jeanjean et al. (2003) *FEBS Letters*, 549, 52–56	IsiA is induced by oxidative stress, suggesting it plays a role in photoprotection

The aim of this article is to provide a detailed review of IsiA research that will help research groups focus their studies on the key issues. Also, it highlights the issues that remain to be addressed.

## Expression and Regulation Of *isia*

### Induction of *isiA* Expression Under Environmental Stresses

A Chl-protein complex, designated CPVI-4, is the major pigment-protein complex in cyanobacteria under iron-starved conditions, which was first described in 1985 (Pakrasi et al., 1985b). Burnap et al. (1993) provided evidence that the CPVI-4 complex was encoded by *isiA*, an iron-stress-induced gene. The *isiA* gene is widely distributed in most cyanobacteria, but no homologs were found in plants (González et al., 2018). It is cotranscribed from the *isiAB* operon containing the *isiB* (flavodoxin gene), *isiC* and *isiD* (unknown functions) in *Synechococcus* sp. PCC 7942, *Synechococcus* sp. PCC 7002, and *Synechocystis* sp. PCC 6803 (Laudenbach and Straus, 1988; Leonhardt and Straus, 1992; Vinnemeier et al., 1998; Kojima et al., 2006). In *Anabaena* sp. PCC 7120, however, *isiA* and *isiB* were found to be separately transcribed (Leonhardt and Straus, 1994). The IsiA protein is often called CP43’ because its amino acid sequence is homologous to that of PsbC, the CP43 protein in cyanobacterial PSII. IsiA, like CP43, is predicted to comprise six transmembrane helices, but it lacks the large hydrophilic loop that joins the luminal ends of helices V and VI in CP43 (Laudenbach and Straus, 1988).

Li et al. (2019) reported that cyanobacterial *IsiA* had a predictable biogeographical distribution in the marine environment, consistent with the perceived biological role of IsiA as an adaptation to low-iron conditions. However, *isiA* transcription was initially discovered in iron-starved cells, it is also found to be induced by other environmental stresses, including salt, heat, oxidative stress, and high levels of light (Vinnemeier et al., 1998; Yousef et al., 2003; Havaux et al., 2005). Vinnemeier et al. (1998) observed that the salt-induced accumulation of *isiA* mRNA was not repressed by the addition of iron; thus, it was unlikely that the salt-dependent induction of *isiA* was due to a reduced iron uptake in the salt-stressed cells. A reasonable explanation for this is that there may be a common signal for the induction of *isiA* generated by salt stress and iron deficiency. Iron deficiency and other stress conditions all eventually lead to a secondary oxidative response (Xu et al., 2014a; Xiao et al., 2018; Yu et al., 2018). Moreover, hydrogen peroxide was found to induce *isiA* transcription much faster than other stresses (Yousef et al., 2003; Dühring et al., 2006); therefore, it is tempting to hypothesize that the oxidative response may be the common downstream signal that regulates *isiA* transcription. In agreement with this hypothesis, *isiA* expression was abolished when iron-stressed cells were grown in the presence of the antioxidant tempol (Latifi et al., 2005). It was also observed that *isiA* transcription was not induced by iron deficiency in cells acclimated to low Mn levels, which had a low PSII activity and decreased electron transport, minimizing the downstream oxidative damage (Salomon and Keren, 2015). This hypothesis explains why *isiA* is expressed in some mutants; for example, the deletion of *psaFJ* or *petJ* resulted in the accumulation of electrons at PSI, with the resulting higher levels of reactive oxygen species triggering the expression of the *isiAB* operon (Ardelean et al., 2002; Jeanjean et al., 2003). The biosynthesis of the IsiA protein was also induced by high light, protecting cyanobacteria from photooxidative stress (Havaux et al., 2005), although the IsiA levels remained much less abundant than in cyanobacteria experiencing iron deficiency.

However, no studies have provided evidence that *isiA* transcription is accompanied by protein biosynthesis under salt, heat, or oxidative stress (Vinnemeier et al., 1998; Hagemann et al., 1999). Mutants lacking *isiA* treated with high salt had only a slightly reduced salt tolerance (Karandashova et al., 2002) and were more resistant to hydrogen peroxide while being more susceptible to sublethal heat stress (Singh et al., 2005; Kojima et al., 2006). This suggests that IsiA functions at an undetectable or very low level under these stress conditions compared to high light and iron deficiency stresses. Indeed, the accumulation of *isiA* mRNA was transiently induced by salt, heat, and oxidative stress, with levels peaking and then immediately decreasing sharply or even disappearing. By contrast, the *isiA* mRNA maximum prevailed for a longer period under high light and iron deficiency stresses (Vinnemeier et al., 1998; Yousef et al., 2003; Dühring et al., 2006). The modification of *isiA* mRNA stability likely also affects the levels of IsiA in cells.

Overall, although the oxidative response could be a secondary signal triggering *isiA* (or *isiAB* operon) transcription, there are some differences in the mechanisms by which iron deficiency stress and oxidative stress (e.g., salt, heat, hydrogen peroxide, and high light stress) induce *isiA* expression. These differences are reflected in the stability of the *isiA* mRNA and ultimately in IsiA accumulation in cells.

### Regulation of *isiA* Expression

The negative regulation of *isiA* is achieved at both the transcriptional and post-transcriptional levels by FurA (ferric uptake regulator A) protein and *isrR* (iron stress-repressed RNA) microRNA, respectively. It is widely accepted that FurA is a global transcription repressor that uses iron as a cofactor, which binds specifically to arrays of A/T-rich sequences known as Fur boxes. Once iron becomes scarce in the environment, FurA is inactivated by the release of iron, triggering the derepression of genes regulated by FurA (Mills and Marletta, 2005). The inactive FurA detaches from the upstream region of the *isiAB* operon; subsequently, the *isiA*/*isiB* genes are transcribed under the iron-deficient conditions. *furA* is an essential gene and was never completely inactivated by insertional mutation; however, heteroallelic *furA* mutants exhibited a blue shift in the main red Chl absorption band under iron-deficient conditions, which is a characteristic symptom of iron deficiency resulting from the appearance of IsiA proteins (Ghassemian and Straus, 1996; Kunert et al., 2003).

Recently, it was reported that the FtsH1/FtsH3 protease heterocomplex mediates the degradation of the FurA repressor and thus promotes IsiA accumulation, which was important for the acclimation of cells to iron deficiency (Krynicka et al., 2014). These results indicate that both the modification of the activity and abundance of FurA influence *isiA* transcription. Other studies have shown that antisense RNAs interfere with the translation of *furA* transcripts, post-transcriptionally affecting the levels of FurA in cyanobacteria (Hernández et al., 2006; Sevilla et al., 2011). Moreover, the *furA* antisense RNA was upregulated as a consequence of oxidative stress (Martin-Luna et al., 2011), suggesting that the induction of *isiA* expression under stress environmental may result from the subsequent oxidative response reducing the level of *furA* mRNA by upregulating its antisense RNA and removing its inhibition of *isiA* expression.

*isrR* is an antisense RNA transcribed from the noncoding strand of *isiA*. Under optimal growth conditions, *isrR* is highly abundant, while *isiA* mRNA is not detectable (Dühring et al., 2006; Xu et al., 2014b). By contrast, *isrR* is degraded and *isiA* mRNA becomes more abundant upon iron deficiency (Dühring et al., 2006), suggesting that the degradation of *isrR* and *isiA* mRNA is linked and could be a reversible switch that responds to iron deficiency. In addition, the degradation of *isrR* was induced by high light and hydrogen peroxide stress (Dühring et al., 2006), indicating another possible mechanism by which *isiA* is induced by various environmental stresses. These stresses caused an oxidative response, which may promote the degradation of *isrR* by unknown regulators and derepress the accumulation of *isiA* mRNA.

Iron deficiency and oxidative stress both stimulate the biosynthesis of IsiA by repressing the levels of *isrR* and FurA; however, iron deficiency has a much greater effect on the induction of *isiA* expression than does oxidative stress. On the one hand, oxidative stress may not effectively repress the activity of FurA mediated by the loss of iron compared to iron-deficient stress itself, although reactive oxygen species can also detach ferrous iron from FurA. On the other hand, this difference could result from the more rapid decrease of *isiA* mRNAs under hydrogen peroxide stress than under iron-deficient stress (Dühring et al., 2006). Mathematical modeling and quantitative experimental analyses showed that *isrR* restricted the accumulation of *isiA* mRNA under prolonged, severe, and unremitting stress conditions, and it was found to be responsible for the rapid decline in *isiA* mRNA levels once the stress was removed (Legewie et al., 2008). Thus, Legewie et al. (2008) speculates that iron deficiency may stimulate a longer oxidative response than oxidative stress in the repression of *isrR*, which maintains the stability of *isiA* mRNA for translation, promoting the accumulation of IsiA protein. Consistent with this hypothesis, IsiA was found to accumulate during the transition from the exponential phase to the stationary phase of growth when PSII photoprotection occurred (Durham et al., 2002; Singh and Sherman, 2006; Foster et al., 2007).

In addition to negative regulation, *isiA* transcription has also been shown to be positively regulated. As a transcriptional repressor, FurA binds to Fur-boxes near the promoter sequences of the *isiA* gene, blocking the entry of RNA polymerase and thereby inhibiting the initiation of transcription. In *Synechocystis* cells, an assay of modified *isiA* promoters fused with GFP showed that only those lacking the Fur motif region did not generate the maximum GFP fluorescence. Kunert et al. (2003) proposed that additional sequence elements in a 90-bp region upstream of the putative −35 box in the *isiA* promoter could be recognized by RNA polymerase, with an unidentified activator affecting its transcriptional activity.

### Model of the Expression and Regulation of *isiA*

**Figure 1:**
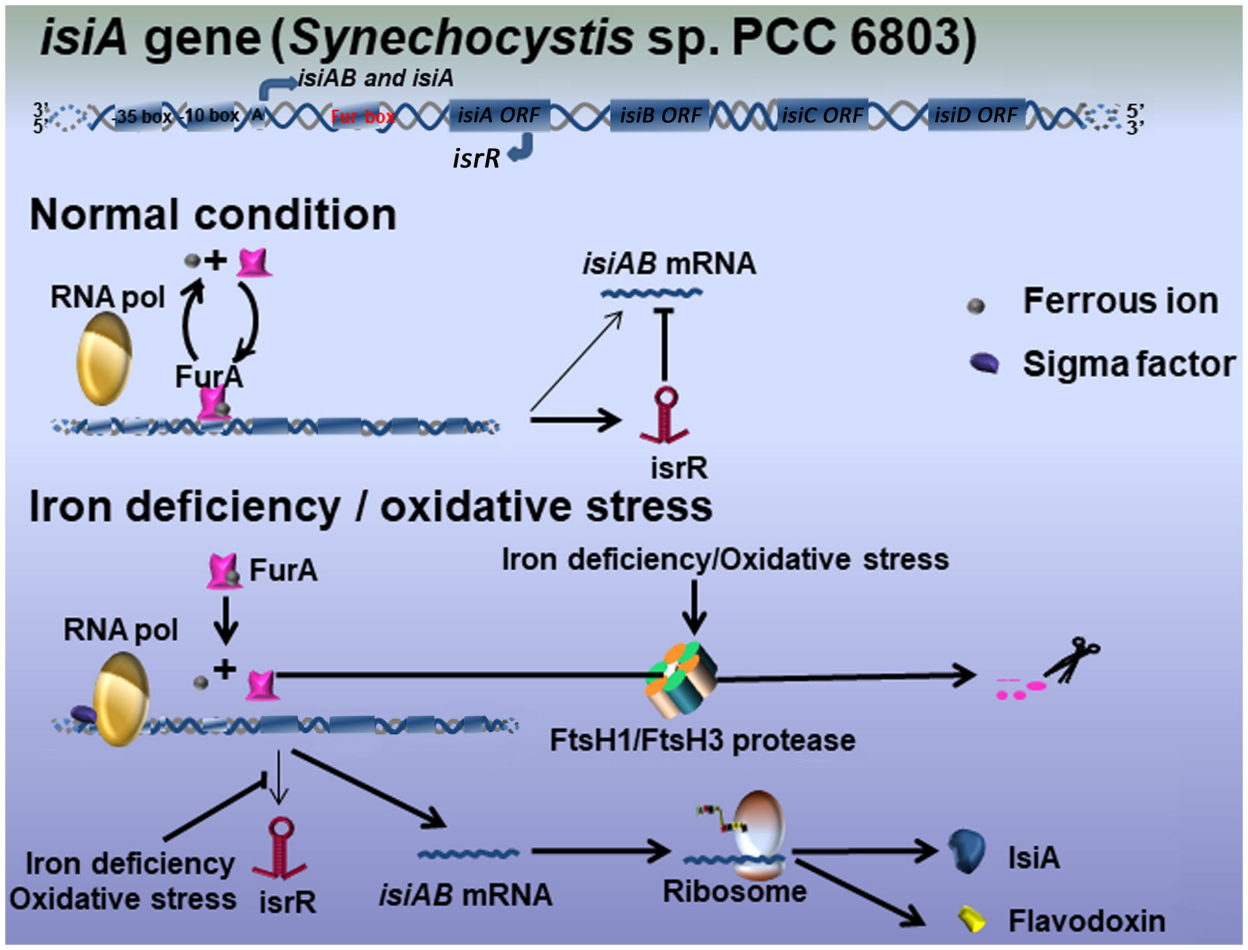
Hypothetical model for the IsiA regulatory mechanisms in *Synechocystis* sp. PCC 6803. Three main transcripts originate in the operon *isiAB*: the bicistronic *isiAB* transcript, the *isiA* monocistronic transcript, and *isrR* microRNA. Under optimal growth conditions, iron-binding FurA represses the expression of *IsiA* at the transcriptional level. The equilibrium between iron-bound and unbound FurA is easily disturbed by variation in the environmental conditions, meaning *isiA* mRNA tends to be continuously expressed at very low levels. The antisense RNA *isrR* mediates the degradation of *isiA* mRNA at the post-transcriptional level. Under iron-limited or oxidative stress conditions, FurA is downregulated by either stress-induced antisense RNA or the FtsH1/FtsH3 protease, leaving RNA polymerase to interact with an unknown activator to initiate *isiA* transcription. *isrR* is also repressed, leading to the further accumulation of *isiA* mRNA and the biosynthesis of IsiA.

The link between iron homeostasis and the redox stress response on the expression of *isiA* highlights the complexity of its regulation, as indicated by multiple studies (Xu et al., 2003; Balasubramanian et al., 2006; Jantaro et al., 2006). The expression and regulation of *isiA* by iron deficiency and oxidative stress may partially overlap. It is probable that the regulation of *isiA* occurs at both the transcriptional and post-transcriptional levels and involves at least two regulators, FurA, the prokaryotic transcriptional regulators that integrate iron metabolism under stress environment, and *isrR*, the only RNA known so far to regulate a photosynthesis component (Dühring et al., 2006; Hernández et al., 2006). A working model is based on studies in *Synechocystis* sp. PCC 6803 (Figure 1). Northern blot analysis showed that more than four different transcripts originated in the *isiAB* operon (Vinnemeier et al., 1998; Dühring et al., 2006): a cis-encoded antisense RNA, *isrR*, transcribed from the *isiA* noncoding strand under optimal growth conditions; the dicistronic *isiAB* transcript; the *isiA* monocistronic transcript; and the 5′ untranslated region transcript (not shown in our model), which was observed but not further analyzed under iron deficiency or oxidative stress.

Briefly, under optimal growth conditions, the iron-binding FurA protein and the *isrR* antisense RNA corepress the biosynthesis of IsiA. It assumed that the equilibrium between iron-bound and nonbound FurA was easily disturbed by variations in environmental conditions, allowing *isiA* to be expressed at very low levels in the cell. It is therefore crucial that *isrR* expression under iron-replete conditions controls the degradation of *isiA* mRNA at the post-transcriptional level. Under iron deficiency or oxidative stress, the level of FurA was downregulated by either stress-induced antisense RNA or FtsH1/FtsH3 protease, allowing RNA polymerase to function in combination with an unknown activator to initiate *isiA* transcription (Krynicka et al., 2014). Under iron-limited conditions, *isrR* expression is repressed by an unknown mechanism, leading to the further accumulation of *isiA* mRNA and IsiA protein (Figure 1). As noted above, it is possible that the effective inactivation of FurA by a loss of iron, as well as the longer oxidative response that represses *isrR* transcription, may be important for the accumulation of IsiA in the cell.

Besides the overlapping regulatory networks mentioned above, it cannot rule out the possibility that iron deficiency stress and other environmental changes or stresses regulate *isiA* expression through different signaling pathways. Lopez-Gomollon et al. (2007) pointed out that nitrogen regulation was present upstream of the *isiA* gene in the N_2_-fixing heterotrophic *Anabaena* sp. PCC 7120. Moreover, Pilla et al. (2013) reported that a heat-responsive transcriptional regulator, Sll1130, might bind to a conserved inverted repeat (GGCGATCGCC) and negatively regulate *isiA* transcription in *Synechocystis* sp. PCC 6803. Thus, additional cis-acting factors might regulate *isiA* transcription to enable cyanobacteria to withstand a variable environment. The model will likely be refined by future research.

### Structure and Function of IsiA Complexes

#### Structural Organization of IsiA Complexes

In 2001, two groups simultaneously reported a supercomplex, IsiA_18_-PSI trimer, consisting of a trimeric PSI surrounded by a closed ring comprising 18 IsiA subunits in two model cyanobacterial species, *Synechocystis* sp. PCC 6803 and *Synechococcus* sp. PCC 7942 (Bibby et al., 2001a; Boekema et al., 2001). Subsequently, electron microscopy and image analyses of IsiA complexes in *Synechocystis* cells under prolonged iron stress conditions revealed a highly flexible interaction between PSI and IsiA or between the IsiA subunits in this complex (Kouril et al., 2005a).

**Figure 2:**
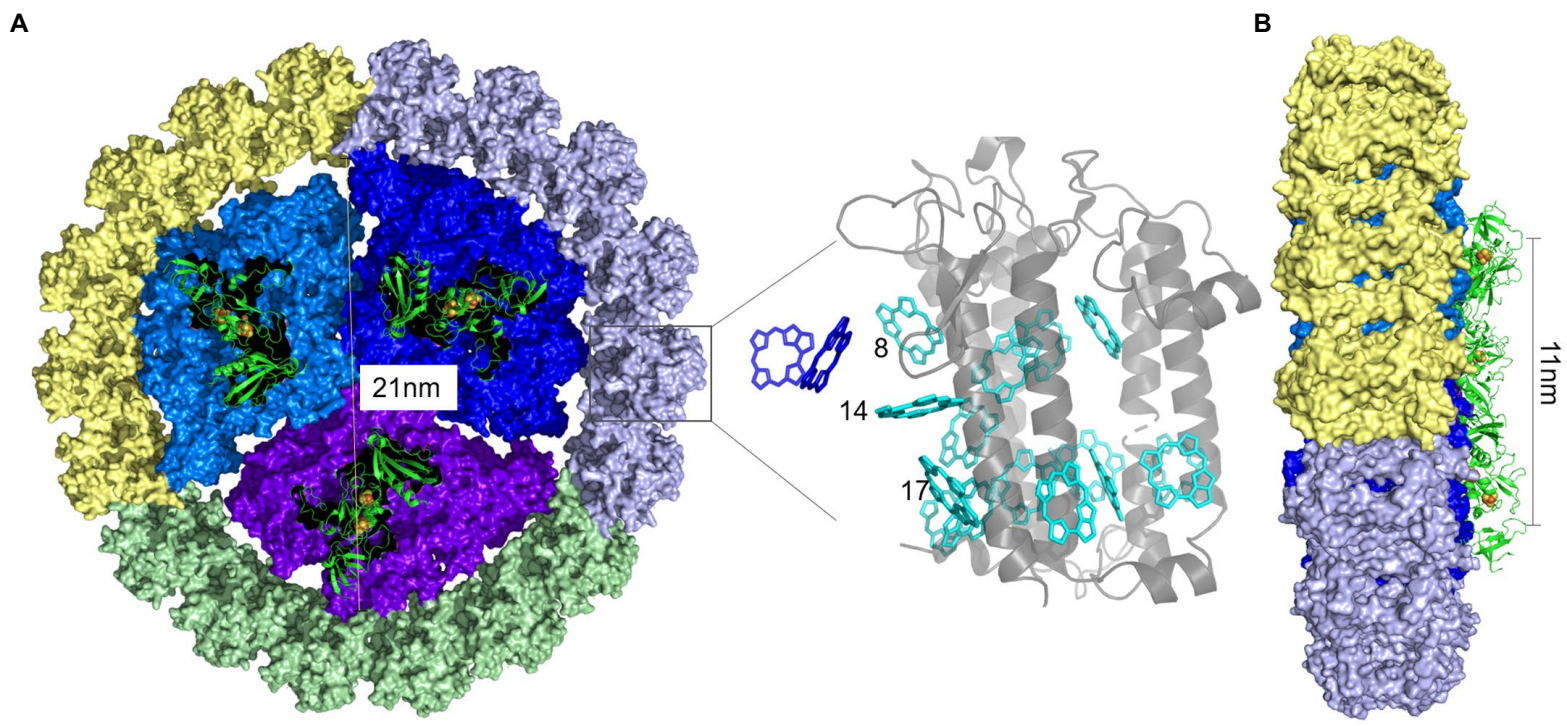
PSI-IsiA supercomplex structure (Drawn according to the study by Toporik et al. (2019) and Cao et al. (2020). A. The IsiA18 –PSI trimer supercomplex structure. 18IsiA monomers form a ring around the PSI trimer. PSI monomers were colored in blue, dark blue and purple, respectively. 6 IsiA proteins surrounding each PSI monomer were colored grey, green and yellow, respectively. The diameter of PSI trimer is 21nm, and the distance between two adjacent highest positions of PSI monomers in PSI trimer is 11nm (B). Chlorophylls around IsiA is cyan, and the Chlorophylls near the PSI trimer is dark blue. Grey is the IsiA protein.

Recently, the overall structure of the IsiA-PSI supercomplex was resolved as a disk of 3-fold rotational symmetry, and the angle is about 120°. The higher-resolution map showed that the supercomplex comprises four helices, each facing PSI, with each IsiA monomer rotated by approximately 60° relative to its neighbor. 18 IsiA monomers form a ring around the PSI trimer, and 6 IsiA monomers around a PSI monomer (Toporik et al., 2019; Cao et al., 2020). In 2020, Zhao et al. had visualized the native organization of the IsiA_18_-PSI trimer in *Synechococcus* sp. PCC 7942 cells by high-resolution atomic force microscopy (Zhao et al., 2020). They found that the diameter of PSI trimer is 21nm, the distance between two adjacent highest positions of PSI monomers in PSI trimer is 11nm (Figure 2). The distance between two adjacent IsiA_18_-PSI trimer supercomplexs is 25.7nm, and the distance between two close IsiA_18_-PSI trimer supercomplexs is 60.2nm (Zhao et al., 2020). They found that IsiA has multiple assembly methods on the thylakoid membrane, which are helical, “S”-shape, strom-like and IsiA fibres insert into the core of an IsiA-PSI supercomplex (Zhao et al., 2020). The typical IsiA_18_-PSI trimer complex is formed during short-term iron-deficient conditions. When the PSI trimers were depolymerized into monomers by prolonged iron deficiency, the IsiA proteins assembled into single rings or double rings surrounding a PSI monomer (Kouril et al., 2005b). Moreover, single, double, triple or multimeric IsiA rings surrounded the PSI trimers, dimers and monomers, and forming IsiA-PSI supercomplex of different structures (Zhao et al., 2020).

Under iron-deficient conditions, the majority of unbound IsiA proteins form IsiA aggregates of different sizes. These IsiA aggregates exist in single rings or double rings, which are similar to the shapes and sizes of those with a central PSI monomer. The existence of the IsiA aggregates demonstrates that the self-assembly of IsiA proteins does not require the presence of either PSI trimers or monomers (Ihalainen et al., 2005). It is often argued that the observed larger IsiA aggregates, with or without PSI monomers, are simply an artifact of severe iron starvation, which leads to chlorosis. By contrast, van der Weij-de Wit et al. (2007) reported that the IsiA aggregates appeared *in vivo* during the early stages of iron stress, which was confirmed by our subsequent finding that the IsiA aggregates formed before the IsiA-PSI trimers (Ma et al., 2017). Furthermore, a sizable pool of uncoupled IsiA aggregates was found in cells grown in a steady-state iron stress condition with replete macronutrients mimicking natural high-nitrate, low-Chl environments (Schrader et al., 2011). By using chlorophyll fluorescence analysis, the IsiA aggregates elevated the Fo (initial fluorescence levels) and decreased the apparent Fv/Fm (the maximum quantum yield of PSII photochemistry), which was consistent with the results of earlier non-steady-state studies (Guikema and Sherman, 1983; Pakrasi et al., 1985a; Falk et al., 1995). Therefore, the significant pool of energetically uncoupled IsiA aggregates is a reality in modern iron-limited environments. Chauhan et al. (2011) observed that the exposure of *Thermosynechococcus elongatus* cells to nanomolar concentrations of iron induced the formation of the largest IsiA-PSI supercomplex, consisting of a PSI trimer surrounded by two complete IsiA rings, with the inner ring comprised of 18 IsiA subunits and the outer ring containing 25 IsiA subunits.

Our previous study suggested that the IsiA proteins seemed to preferentially encircle trimeric PSI when both the PSI trimers and monomers were present in cells exposed to short-term iron deficiency (Ma et al., 2017). The binding preference of IsiA is similar to IdiA, another iron-stress-induced protein that is not homologous to IsiA and binds only to dimeric, not monomeric, PSII (Lax et al., 2007); however, the trimeric organization of PSI was not necessary for the binding of the IsiA protein, as noted above. Moreover, IsiA still had a strong tendency to form complexes with the PSI monomer in a *psaL* deletion mutant lacking PSI trimers, even in short-term iron-deficient conditions (Aspinwall et al., 2004; Kouril et al., 2005b). In the *psaL* deletion mutant, incomplete single or double rings of IsiA proteins specifically bound next to the PsaF/J subunits in the PSI monomer, indicating that these subunits might be responsible for the binding of IsiA. Interactions were observed between IsiA’s “a” “c” “d” “e” positions and PSI subunits PsaF, PsaJ, and PsaK, revealing that these protein–protein interactions involved the C terminus of IsiA (Figure 2; Toporik et al., 2019). Nevertheless, in the mutant strain lacking the PsaF and PsaJ subunits, IsiA was still capable of binding the PSI trimer as a ring of 17units (Kouřil et al., 2003). These results clarified that the PsaL and PsaF/J subunits facilitated the binding of IsiA to PSI but were not obligatory structural components in the formation of the IsiA-PSI complexes. The observation that IsiA proteins specifically associate with PSI monomers next to the PsaF/J subunits suggests that the IsiA docking site is located near these subunits. In plants, PsaG acts as a linker protein that anchors a belt of four light-harvesting proteins, Lhca1–4, to the side of the PsaF/J subunits of the PSI core complex *via* its two tilting transmembrane helices (Ben-Shem et al., 2003). In cyanobacteria, an accessory protein factor similar to PsaG plays a role in the assembly of the IsiA-PSI complex, but whether it is absent in the resulting complex is unknown (Boichenko, 2004). Schoffman and Keren (2019) demonstrated that the biodilution of intercellular Fe was the main factor that controlled the formation of IsiA pigment–protein complexes.

There are 591 Chls on IsiA_18_-PSI trimer supercomplex, 306 Chls with the IsiA ring and 298 Chls in PSI trimer in *Synechocystis* sp. PCC 6803. However, in different cyanobacterium, the IsiA_18_-PSI trimer supercomplex combines different Chl a and Car, such as the IsiA_18_-PSI trimer of *Synechococcus* sp. PCC 7942 binds to 3 Chl a and 3 Car more than *Synechocystis* sp. PCC 6803 in the core part (Toporik et al., 2019; Cao et al., 2020). Feng et al. (2011) have reported that IsiA most likely bound to 13 Chls, in agreement with the number of Chls in CP43 of PS II. Toporik et al. (2019) pointed out each IsiA monomer bound to 17 Chls, of which 13 are in a similar position to their location on CP43 and four are specific to IsiA. Ihalainen et al. (2005) showed that four Cars were present in each IsiA monomer, including two β-carotenoids, one echinaceone, and one zeaxanthin. However, Toporik et al. (2019) identified three Cars at the IsiA-IsiA interface, and another Car, B1, bound to the interface between IsiA and PSI. Cao et al. (2020) reported the structures of the PSI_3_–IsiA_18_–Fld_3_ and PSI_3_–IsiA_18_ supercomplexes from *Synechococcus sp*. PCC 7942, revealing features that are different from the previously reported PSI structures, and a sophisticated pigment network that involves previously unobserved pigment molecules. Pigment analysis results showed that, when compared with the trimeric PSI core alone, the PSI_3_–IsiA_18_ supercomplex contains higher amounts of zeaxanthin (Zea), a molecule that is essential for quenching excessive absorbed energy.

The prochlorophytes, such as the marine phytoplankton *Prochlorococcus*, are a class of cyanobacteria that do not use phycobilisomes, but instead use intrinsic light-collecting proteins, known as Pcb proteins, which contain Chl a/b as light-harvesting systems. There are six transmembrane helices, and highly homology with the chlorophyll-binding protein that includes the Chl a-binding proteins CP43, CP47 of PSII and the iron stress-induced protein IsiA. Some constitutively expressed Pcbs form light-harvesting structures in *Prochlorococcus* strains, including the PcbG_18_-PSI trimer in the low-light-adapted strain SS120, the PcbA_8_-PSII dimer in the moderate low-light-adapted strain *Prochlorococcus* sp. MIT9313 and in the high-light-adapted strain MED4 (Bibby et al., 2003). In *Prochlorococcus* sp. MIT 9313, iron deficiency could induce the formation of PcbB_18_-PSI trimer complexes similar to the IsiA_18_-PSI trimer (Bibby et al., 2003). MED4 PSI domains were loosely packed in the thylakoid membrane of *Prochlorococcus* sp. MIT 9313, while in the low-light condition, PSI was organized into a tightly packed pseudo-hexagonal lattice to maximize harvesting and trapping of light. The low-light-adapted algal strain SS120 has a different strategy for coping with low-light levels, and SS120 thylakoids contained hundreds of tightly packed Pcb-PSI supercomplexes, saving the extra iron and nitrogen required to PSI-only domains (MacGregor-Chatwin et al., 2019). These results indicated that it was a common phenomenon to express iron-deficiency induced antenna pigment protein and assemble to maintain photosynthesis under iron deficiency conditions.

#### Diversity of IsiA Function

As mentioned above, IsiA shares high sequence similarity with CP43 in PSII but lacks the large hydrophilic loop between helices V and VI of CP43 (Chen and Bibby, 2005). In *Synechocystis* sp. PCC 6803, deletion mutations in the long hydrophilic loop of CP43 led to the disappearance of oxygen evolution activity (Kuhn and Vermaas, 1993), suggesting that IsiA cannot functionally replace CP43 under iron-deficient conditions, as was proposed by Burnap et al. (1993). Using mutants lacking the long hydrophilic loop of CP43, it was confirmed that no detectable differences were present in the light-dependent evolution of oxygen under iron deficiency and salt stress conditions (Vinnemeier et al., 1998). A high-resolution crystal structure of PSII at a resolution of 1.9Å showed that the CP43-Glu 354 residue was located in the hydrophilic loop in the Mn_4_CaO_5_ cluster of the water-splitting reaction center, where it functions as a bidentate ligand for Mn_2_ and Mn_3_ (Umena et al., 2011). As the loss of the hydrophilic loop leads to the loss of hydrolysis ability, IsiA cannot replace CP43.

When iron becomes readily available in the environment, iron-starved cells recover quickly, resynthesizing the thylakoid membranes and reassembling their Chl-containing protein complexes (Sherman and Sherman, 1983; Odom et al., 1993). Using a Chl biosynthesis inhibitor, some studies have shown that the reassembly of Chl-containing protein complexes occurred before Chl biosynthesis (Riethman and Sherman, 1988; Troyan et al., 1989). IsiA is the major Chl-containing protein, binding up to 50% of the Chl in iron-starved cells (Burnap et al., 1993). In iron-limited cultures, transfer of half of the chlorophyll in the cell from PSI to IsiA had negligible effects on energy transfer from antenna systems to PSII *in vivo* (Schoffman and Keren, 2019). Therefore, the IsiA pigment-protein complex not only plays a key role when cells transition into iron limitation, but also supports the efficient recovery of photosynthetic apparatus during cells transition back out of iron limit-phase (Schoffman and Keren, 2019). However, the above content may not be the main function of IsiA, it should only be a cost-effective way of supplying preexisting Chl in IsiA for the reassembly of PSII and PSI complexes after the addition of iron.

Due to its high level of Chl binding, IsiA was also hypothesized to serve as an alternative light-harvesting complex, compensating for the decrease in phycobilisomes in iron-starved cells (Burnap et al., 1993; Schoffman and Keren, 2019). Cheng et al. (2020) showed the deletion of *isiA* in *Synechocystis* sp. PCC 6803 affected the genes expression that are involved in photosynthesis, phycobilisome, and the proton-transporting ATPase complex. A 77K fluorescence spectrum revealed that the characteristic peak of isolated IsiA aggregates at around 685nm was very high but was weak in the IsiA_18_-PSI trimer complex (Bibby et al., 2001a). Furthermore, treating the IsiA_18_-PSI trimer complex with Triton X-100 caused this weak peak to become the dominant emission (Bibby et al., 2001b), indicating that PSI trimer coupling IsiA in such a way is to efficiently transfer light energy to the PSI reaction center. In another report, Toporik et al. (2019) proposed that the large amount of chlorophyll in the stromal layer meant that photons would be absorbed by this layer and then converted into excitation energy to be transferred to PSI through the luminal layer. Chlorophyll at position 17 (chlorophyll 17) through chlorophyll at position 8 (chlorophyll 8) combined with the main pigment cluster, with chlorophyll 8 being unique to IsiA (Figure 2). The specific position of chlorophyll 17 and 8 molecules showed that they were the terminal emitters of IsiA and thus played a key role in the process of energy transfer. More chlorophyll was distributed on the stromal side of the membrane, where it formed a continuous pigment layer around PSI, which is different from what was observed in eukaryotes; therefore, Toporik et al. (2019) speculated that this phenomenon plays an important role in the photoprotective process.

Using different spectroscopic measurements, such as time-resolved absorption and emission spectroscopy, the energy transfer and trapping processes in the IsiA_18_-PSI trimers in *Synechocystis* sp. PCC 6803, *Synechococcus* sp. PCC 7942 and *Thermosynechococcus vulcanus* were studied (Andrizhiyevskaya et al., 2002; Melkozernov et al., 2003; Andrizhiyevskaya et al., 2004; Akita et al., 2020). For the largest IsiA_43_-PSI supercomplex, identified in *T. elongatus*, fluorescence-decay-associated spectra also indicated that IsiA was energetically strongly associated with the PSI trimer (Chauhan et al., 2011). Based on calculations of the optimal energy transfer within the IsiA_18_-PSI trimer, IsiA was proposed to bind 15 Chls in *Synechocystis* sp. PCC 6803 (Zhang et al., 2010); however, Feng et al. (2011) showed that IsiA most likely possessed 13 Chls in *Synechocystis* sp. PCC 6803 in agreement with the number in CP43 of PSII, as determined from a crystal structure with a resolution of 1.9Å. Recently, Toporik et al. (2019) pointed out each IsiA monomer bound to 17 Chls. As 96 Chl molecules are present in the PSI monomer, determined from a crystal structure with a higher resolution of 2.5Å (Jordan et al., 2001), the theoretical cross sections of the IsiA_18_-PSI complex should increase by approximately 81% compared to the PSI trimer alone (Bibby et al., 2001a). Using a light saturation curve in *Synechococcus* sp. PCC 7942, the light-harvesting ability of the IsiA_18_-PSI trimer was only found to be about 44% higher than that of PSI (Boekema et al., 2001). In addition to *in vitro* measurements, Ryan-Keogh et al. (2012) demonstrated an increase of 60% in the absorption cross section of PSI in iron-starved *Synechocystis* sp. PCC 6803 cells. Another report provided evidence that the increased absorption cross section provided by the IsiA proteins led to an enhanced rate of electron transfer through PSI in the marine strain *Synechococcus* sp. PCC 7002 (Sun and Golbeck, 2015).

In the IsiA_18_-PSI trimer structural model, the 18 IsiA proteins did not form a perfect ring and were instead distorted by the 3-fold rotational symmetry of the PSI trimer (Nield et al., 2003; Feng et al., 2011). Only the regions where PSI Chl a molecules are located close to the IsiA Chl a molecules were believed to be involved in transferring energy from IsiA to the PSI trimer. It was also proposed that not all IsiA proteins transfer energy directly to the inner PSI molecules, due to their nonequivalent positions relative to PSI. Akita et al. (2020) recently report a 2.7-Å resolution cryo-electron microscopic structure of a supercomplex between PSI core trimer and IsiA from a thermophilic cyanobacterium *Thermosynechococcus vulcanus*, and time-resolved fluorescence spectra of the IsiA_18_-PSI trimers supercomplex showed clear excitation-energy transfer from IsiA to PSI, strongly indicating that IsiA functions as an energy donor, but not an energy quencher, in the supercomplex.

Toporik et al. (2019) demonstrated that the crystal structure of IsiA has been resolved, the Chl in the IsiA loop is unequally distributed, and the stromal side of the membrane contains nearly twice as many pigments as the lumenal side. In the PSI-IsiA supercomplex at the stromal side, only one chlorophyll pair is located close to 18Å, and the next pair is at 21–25Å. These distances are enough to mediate energy transfer, but much larger than most Chl distances between internal IsiA and PSI, further indicating that the IsiA loop and PSI are independently present on the stromal side. On the luminal side, where there is little pigment, 10 Chl pairs were observed below 20Å, linking IsiA and PSI, most of which involved Chl 17, 14, and 8 on the IsiA subunit (Figure 2). Due to their abundance in the stromal layer, photons are more likely to be absorbed by the stromal layer, but the excitation energy is more likely to be transferred to the PSI through the lumenal side. The location of Chl 8 and 17 at the interface between the adjacent IsiA subunit and PSI strongly indicates that these Chl molecules are terminal emitters of IsiA and should play a key role in their function (Toporik et al., 2019).

In addition, steady-state and time-resolved fluorescence measurements indicated that isolated IsiA aggregates dissipated excitation energy, after which they were in a strongly fluorescence-quenched state (Ihalainen et al., 2005). The IsiA aggregates are present in the early stages of iron starvation, and their fluorescence quenching is similar to that observed under long-term iron starvation (van der Weij-de Wit et al., 2007). Therefore, IsiA might mediate the thermal dissipation of absorbed energy and thereby protect PSII from excessive excitation under iron-limited conditions, as predicted in previous studies (Park et al., 1999; Sandström et al., 2001). It is well established that Car can quench the excited state of Chl, and a similar mechanism appears to operate in IsiA aggregates (Berera et al., 2009); for example, a high-performance liquid chromatography analysis revealed that IsiA aggregates contain Chl a, β-carotene, echinenone, and zeaxanthin (Ihalainen et al., 2005; Berera et al., 2009). In a mutant strain lacking *CrtO*, which encodes β-carotene ketolase, the enzyme mediating the conversion of β-carotene to echinenone, the IsiA aggregates lacking echinenone were not deficient in their fluorescence quenching ability (Dhaene et al., 2008).

#### Model of the Structures and Functions of IsiA Complexes

Under iron-replete conditions, the PSI/PSII ratio is high and sufficient phycobilisomes can move between the two photosynthetic systems, which together maintain an electron flow balance between PSI and PSII. Under iron-limited conditions, the PSI/PSII ratio drops and the phycobiliprotein content is significantly reduced, perturbing the electron balance between the photosystems and resulting in serious oxidative damage. In the electron transport chain, PSII is more labile and vulnerable to oxidative damage (Allakhverdiev et al., 2008). The importance of defending PSII from oxidative damage under iron-deficient conditions has been confirmed based on several observations, including the rapid increase in thermal dissipation at the level of the antenna associated with an orange carotenoid protein (OCP; Wilson et al., 2006; Wilson et al., 2007) and the induction of another iron stress protein, IdiA, that protects the acceptor side of PSII against photodamage (Exss-Sonne et al., 2000; Lax et al., 2007). The transcription of *idiA* precedes that of *isiA*, and the deletion of *idiA* promotes the formation of IsiA-PSI complexes under iron starvation (Tölle et al., 2002; Yousef et al., 2003), indicating that the expression of *isiA* cannot be effectively prevented when PSII is damaged.

Recently, using immunoblot and 77K fluorescence analyses performed with thylakoid membranes and fractions from a sucrose gradient ultracentrifugation, we observed that free IsiA proteins preferentially encircled the PSI trimer to efficiently transfer energy to the PSI cores, even without an IsiA-originated fluorescence peak, a state of IsiA-PSI trimer that had not previously been reported (Ma et al., 2017). IsiA-PSI complexes formed and gradually accumulated throughout the iron deficiency period, providing more convincing evidence that the original role of IsiA bound to PSI was as an energy collector for PSI. Not all energy absorbed by IsiA is transferred to PSI, especially during prolonged iron deficiency, possibly because the increased *in vivo* cross section of PSI is lower than the theoretical increased cross section of the IsiA_18_-PSI trimer (Ma et al., 2017). As already noted, the IsiA aggregates likely appeared before the formation of the IsiA_18_-PSI trimer and became larger following prolonged iron deficiency, dissipating excess energy throughout the course of iron deficiency.

**Figure 3:**
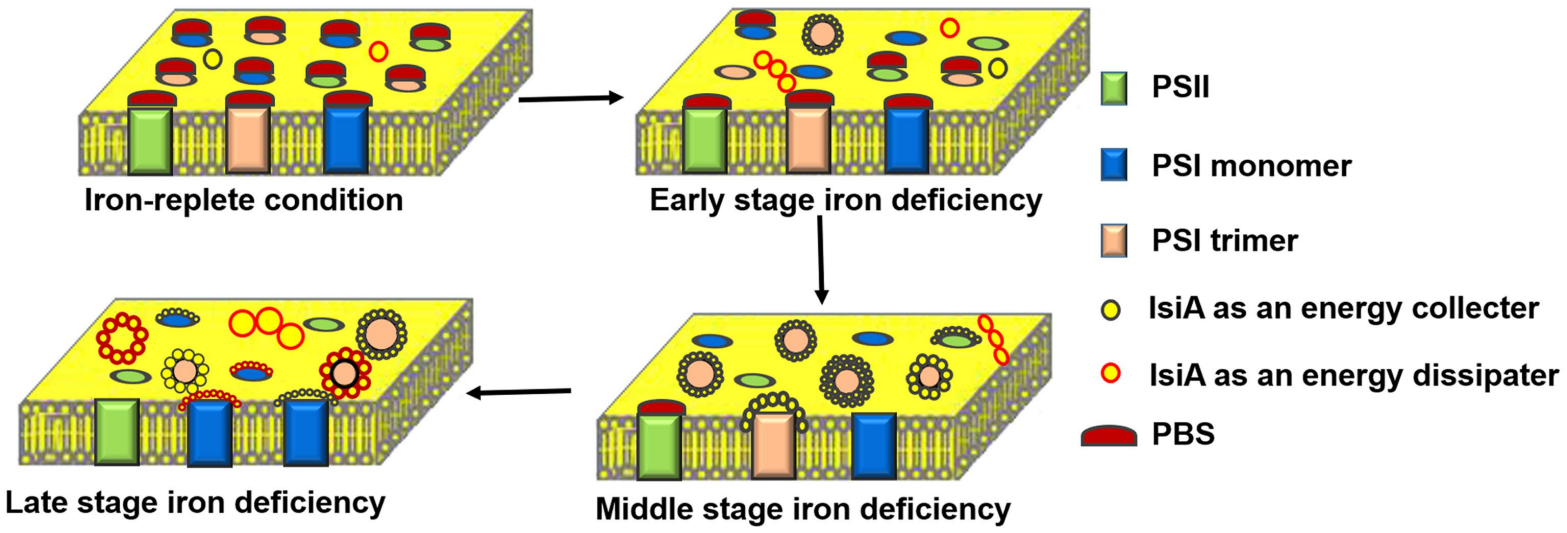
Schematic representation of the dynamic changes in the structure and function of IsiA-containing complexes during a long period of iron deficiency. Under the iron-replete condition, phycobilisome (PBS) acts as a light-harvesting complex for both PSII and PSI. In the early stages of iron deficiency, IsiA forms small aggregates and IsiA_18_-PSI trimers, in which the IsiA proteins serve as energy collectors and efficiently transfer energy to the PSI cores. In the middle stage of iron deficiency, the levels of PSB, PSI, and PSII all decline, with PSB still serving PSII. An IsiA-PSI low fluorescence supercomplex (ILFS) is formed, in which IsiA acts an energy collector. In the late stage of iron deficiency, the IsiA-PSI high fluorescence supercomplex (IHFS) and plentiful IsiA aggregates are formed. The IsiA proteins serve as an energy dissipater, providing photoprotection for the entire photosynthetic apparatus, especially PSII.

Thus, a scenario for the dynamic change in IsiA complex structures and the roles of IsiA in these complexes during long-term periods of iron deficiency under laboratory conditions was presented (Figure 3). In the early stages of iron-limited stress, the IdiA proteins are produced and located at the acceptor side of PSII, where they prevent photodamage. In the middle stage, the IsiA proteins possibly appear as small aggregates, but later begin to associate with the PSI trimer to efficiently transfer energy to the PSI cores by IsiA-PSI supercomplex. In the late stage of iron-limited stress, the PSI trimers depolymerize to monomers, leaving the IsiA proteins to be incorporated into larger IsiA aggregates or form IsiA-PSI monomer complexes, in which the IsiA proteins have the primary function of being energy collectors and the secondary function of dissipating energy to provide photoprotection for PSI. Chen et al. found that IsiA quench excitation energy by a novel cysteine-mediated process for the photoprotection (Chen et al., 2021). The IsiA aggregates dissipate excess energy, providing photoprotection for the whole photosynthetic apparatus, especially PSII. The dynamic changes in IsiA and its associations with PSI and PSII may be an optimal adaptation to the degree of iron deficiency, enabling flexible light harvesting and balancing electron transfer between PSI and PSII to minimize photodamage, and ensuring the cells survive. By contrast, Toporik et al. (2019) observed that, besides the light-harvesting and photoprotection functions of IsiA, the IsiA dimer can also maintain the effective transfer of excitation energy in the IsiA ring.

The function of the IsiA−PSI supercomplex that favour energy transfer towards the central PSI was confirmed recently by structure analysis of membrane complexes in the cyanobacterial thylakoid membrane using high-resolution atomic force microscopy (Zhao et al., 2020). However, although significant progress has been made in our understanding of the IsiA complexes (Figure 3), many questions remain, and further verification is required. For example, whether IsiA and PSII can associate with each other is still under debate. It was clear that accumulated IsiA did not contribute to light capture by PSII (Falk et al., 1995; Fraser et al., 2013); however, energy transfer was observed between PBS and the IsiA aggregates (Rakhimberdieva et al., 2007; Wilson et al., 2007). This supports the proposal that IsiA displays a remarkable mobility (Sarcina and Mullineaux, 2004), enabling it to be located between PSII and PBS within the membrane and thereby compete with the energy transfer from PBS to PSII (or PSI; Huner et al., 2001). Notably, Toporik et al. (2019) demonstrated that the IsiA ring interacted with soluble PBS. Using BN-SDS-PAGE, immunoblot, and 77K fluorescence analyses, Wang et al. (2010) demonstrated that a IsiA-PSI-PSII supercomplex was present in long-term iron-starved cells, which is inconsistent with the result that no potential structural information about the IsiA-PSII complex was detected under long-term iron-deficient conditions using electron microscopy (Kouril et al., 2005a).

Within the IsiA-PSI-PSII supercomplexes, IsiA was proposed to be a regulator or assembler, rather than physically encircling the complexes. In addition, it was previously shown that the IsiA protein was enriched in HLIP (high light inducible protein)-containing PSI trimers prepared from *Synechocystis* sp. PCC 6803 cells treated with high-intensity light (Wang et al., 2008). Further studies using a *PsaL*-deleted mutant lacking the PSI trimers showed that IsiA was a component of the novel high-light-inducible carotenoid-binding protein complex (HLCC), consisting of Slr1128, IsiA, PsaD, and high-light-induced proteins A/B (HliA/B; Daddy et al., 2015). In the latter study, the authors hypothesized that the HLCC is also induced by iron deficiency and oxidative stress. In this case, IsiA, together with other components, might be localized at the stromal side of PSI, mediated by PsaD, to stabilize trimeric PSI and protect PSI from direct or indirect oxidative stresses, probably by scavenging reactive oxygen species produced at ferredoxin (Fd). The assembly of IsiA in the HLCC appeared to be a more accepted explanation of the role of IsiA under oxidative stress, despite its much lower abundance under oxidative stress than iron-deficient stress. The revelation of the IsiA-PSI-PSII supercomplex and HLCC further expands our understanding of the structure and function of the IsiA complexes.

## Conclusion and Prospects

Over the almost 30years since its discovery, there has been tremendous progress in our understanding of the iron-stressed *isiA* gene. The induction of *isiA* expression by various unfavorable environmental stresses or abnormal physiological states (such as in genetic mutants) reflects the complexity of the regulation of *isiA* expression. While the oxidative response could be considered a superior downstream trigger for *isiA* transcription, it is not enough to strongly induce the translation of IsiA proteins. Therefore, the regulation of IsiA function occurs at both the transcriptional and translational levels, involving at least FurA and the *isrR* antisense RNA, respectively. Moreover, it is likely that the effective inactivity of FurA by the loss of iron and the stabilization of *isiA* mRNA by the repression of *isrR* expression following an extended oxidative response are both closely related to the accumulation of IsiA proteins in cyanobacteria.

As described above, the model of the regulation of *IsiA* expression should be improved upon in the future. Physiological and functional analyses demonstrated that IsiA acts as an energy collector or an energy dissipater, depending on the functions of the complexes it forms. While structural models of the IsiA_18_-PSI trimer have been presented, a high-resolution crystal structure of this complex would be helpful for determining which domains and subunits of PSI are crucial for IsiA binding and which Chls are crucial for the energy transfer network. Similarly, further studies are required to establish whether zeaxanthin or β-carotene acts as an energy quencher in these complexes or if another factor is involved. Moreover, the factors that initiate or facilitate the binding of IsiA to the PSI trimer are not currently known and should be identified. In addition, the existence of the IsiA-PSI-PSII supercomplex is yet to be verified, and further studies of HLCC will greatly enhance our knowledge of the physiological functions of IsiA.

## Author Contributions

AJ, YZ, and QW conceptualized the idea for manuscript. AJ, YZ, and HC drafted the manuscript. AJ and YZ made the figures. QW evaluated the manuscript and improvised the content. All authors contributed to the article and approved the submitted version.

## Funding

This work was supported jointly by the National Key Research and Development Program of China (2020YFA0907600), the National Natural Science Foundation of China (31,770,128, 91,851,103 and 31,870,041), and the Natural Science Foundation of Henan Province (212300410024).

## Conflict of Interest

The authors declare that the research was conducted in the absence of any commercial or financial relationships that could be construed as a potential conflict of interest.

## Publisher’s Note

All claims expressed in this article are solely those of the authors and do not necessarily represent those of their affiliated organizations, or those of the publisher, the editors and the reviewers. Any product that may be evaluated in this article, or claim that may be made by its manufacturer, is not guaranteed or endorsed by the publisher.

## References

[ref1] Akita F. Nagao R. Kato K. Nakajima Y. Yokono M. Ueno Y.. (2020). Structure of a cyanobacterial photosystem I surrounded by octadecameric IsiA antenna proteins. Communications Biology 3, 232. doi: 10.1038/s42003-020-0949-6, PMID: 32393811 PMC7214436

[ref2] Allakhverdiev S. I. Kreslavski V. D. Klimov V. V. Los D. A. Carpentier R. Mohanty P. (2008). Heat stress: an overview of molecular responses in photosynthesis. Photosynth Research 98, 541–550. doi: 10.1007/s11120-008-9331-0, PMID: 18649006

[ref3] Andrizhiyevskaya E. G. Frolov D. Van Grondelle R. Dekker J. P. (2004). Energy transfer and trapping in the photosystem I complex of *Synechococcus* PCC 7942 and in its supercomplex with IsiA. Biochim. Biophys. Acta 1656, 104–113. doi: 10.1016/j.bbabio.2004.02.002, PMID: 15178472

[ref4] Andrizhiyevskaya E. G. Schwabe T. M. Germano M. D'Haene S. Kruip J. van Grondelle R.. (2002). Spectroscopic properties of PSI–IsiA supercomplexes from the cyanobacterium *Synechococcus* PCC 7942. Biochimica et Biophysica Acta (BBA)-Bioenergetics 1556, 265–272. doi: 10.1016/S0005-2728(02)00371-7, PMID: 12460685

[ref5] Ardelean I. Matthijs H. Havaux M. Joset F. Jeanjean R. (2002). Unexpected changes in photosystem I function in a cytochrome c6-deficient mutant of the cyanobacterium *Synechocystis* PCC 6803. FEMS Microbiol. Lett. 213, 113–119. doi: 10.1111/j.1574-6968.2002.tb11294.x, PMID: 12127497

[ref6] Aspinwall C. L. Duncan J. Bibby T. Mullineaux C. W. Barber J. (2004). The trimeric organisation of photosystem I is not necessary for the iron-stress induced CP43 protein to functionally associate with this reaction Centre. FEBS Lett. 574, 126–130. doi: 10.1016/j.febslet.2004.08.016, PMID: 15358552

[ref7] Balasubramanian R. Shen G. Bryant D. A. Golbeck J. H. (2006). Regulatory roles for IscA and SufA in iron homeostasis and redox stress responses in the cyanobacterium *Synechococcus* sp. strain PCC 7002. Journal of Bacteriol 188, 3182–3191. doi: 10.1128/jb.188.9.3182-3191.2006, PMID: 16621810 PMC1447454

[ref8] Behrenfeld M. J. Milligan A. J. (2013). Photophysiological expressions of iron stress in phytoplankton. Annu. Rev. Mar. Sci. 5, 217–246. doi: 10.1146/annurev-marine-121211-172356 22881354

[ref9] Ben-Shem A. Frolow F. Nelson N. (2003). Crystal structure of plant photosystem I. Nature 426, 630–635. doi: 10.1038/nature02200, PMID: 14668855

[ref10] Berera R. van Stokkum I. H. d'Haene S. Kennis J. T. van Grondelle R. Dekker J. P. (2009). A mechanism of energy dissipation in cyanobacteria. Biophys. J. 96, 2261–2267. doi: 10.1016/j.bpj.2008.12.3905, PMID: 19289052 PMC2717300

[ref11] Bibby T. Mary I. Nield J. Partensky F. Barber J. (2003). Low-light-adapted *Prochlorococcus* species possess specific antennae for each photosystem. Nature 424, 1051–1054. doi: 10.1038/nature01933, PMID: 12944966

[ref12] Bibby T. S. Nield J. Barber J. (2001a). Iron deficiency induces the formation of an antenna ring around trimeric photosystem I in cyanobacteria. Nature 412, 743–745. doi: 10.1038/35089098, PMID: 11507643

[ref13] Bibby T. S. Nield J. Barber J. (2001b). Three-dimensional model and characterization of the iron stress-induced CP43′-photosystem I supercomplex isolated from the cyanobacterium *Synechocystis* PCC 6803. J. Biol. Chem. 276, 43246–43252. doi: 10.1074/jbc.M106541200 11518716

[ref14] Boekema E. Hifney A. Yakushevska A. Piotrowski M. Keegstra W. Berry S.. (2001). A giant chlorophyll–protein complex induced by iron deficiency in cyanobacteria. Nature 412, 745–748. doi: 10.1038/35089104, PMID: 11507644

[ref15] Boichenko V. A. (2004). Photosynthetic units of phototrophic organisms. Biochemistry-Moscow 69, 471–484. doi: 10.1023/b:biry.0000029844.31857.40, PMID: 15193120

[ref16] Burnap R. L. Troyan T. Sherman L. A. (1993). The highly abundant chlorophyll-protein complex of iron-deficient *Synechococcus* sp. PCC7942 (CP43 [prime]) is encoded by the isiA Gene. Plant Physiol. 103, 893–902. doi: 10.1104/pp.103.3.893, PMID: 8022940 PMC159061

[ref17] Cao P. Cao D. F. Si L. Su X. D. Tian L. J. Chang W. R.. (2020). Structural basis for energy and electron transfer of the photosystem I-IsiA-flavodoxin supercomplex. Nature Plants 6, 167–176. doi: 10.1038/s41477-020-0593-7, PMID: 32042157

[ref18] Catling D. C. Zahnle K. J. (2020). The Archean atmosphere. Science advances 6:16. doi: 10.1126/sciadv.aax1420 PMC7043912 32133393

[ref19] Chauhan D. Folea I. M. Jolley C. C. Kouril R. Lubner C. E. Lin S.. (2011). A novel photosynthetic strategy for adaptation to low-iron aquatic environments. Biochemistry 50, 686–692. doi: 10.1021/bi1009425, PMID: 20942381

[ref20] Chen H. Niedzwiedzki D. M. Bandyopadhyay A. Biswas S. Pakrasi H. B. (2021). A Novel Mode of Photoprotection Mediated by a Cysteine Residue in the Chlorophyll Protein IsiA. *microbiology* 12:14. doi: 10.1128/mBio.03663-20. PMC8545134 33593975

[ref21] Chen M. Bibby T. S. (2005). Photosynthetic apparatus of antenna-reaction centres supercomplexes in oxyphotobacteria: insight through significance of Pcb/IsiA proteins. Photosynth. Res. 86, 165–173. doi: 10.1007/s11120-005-1330-9, PMID: 16172936

[ref22] Cheng Y. R. Zhang T. Y. Wang L. Chen W. L. (2020). Transcriptome analysis reveals IsiA-regulatory mechanisms underlying iron depletion and oxidative-stress acclimation in Synechocystis sp. strain PCC 6803. Applied and environmental microbiology. 86. doi: 10.1128/AEM.00517-20, PMID: 32332138 PMC7301839

[ref23] Daddy S. Zhan J. Jantaro S. He C. He Q. Wang Q. (2015). A novel high light-inducible carotenoid-binding protein complex in the thylakoid membranes of *Synechocystis* PCC 6803. Sci. Rep. 5. doi: 10.1038/srep09480, PMID: 25820628 PMC4377637

[ref24] Dhaene S. Tsoukatos K. Lampoura S. S. Matthijs H. C. Dekker J. P. (2008). “role of echinenone in fluorescence quenching in IsiA aggregates from cyanobacteria,” in photosynthesis. Energy from the Sun. Springer. 253–256.

[ref25] Dühring U. Axmann I. M. Hess W. R. Wilde A. (2006). An internal antisense RNA regulates expression of the photosynthesis gene isiA. Proc. Natl. Acad. Sci. 103, 7054–7058. doi: 10.1073/pnas.0600927103, PMID: 16636284 PMC1459017

[ref26] Durham K. A. Porta D. Twiss M. R. McKay R. M. L. Bullerjahn G. S. (2002). Construction and initial characterization of a luminescent *Synechococcus* sp. PCC 7942 Fe-dependent bioreporter. FEMS Microbiol. Lett. 209, 215–221. doi: 10.1016/s0378-1097(02)00567-0, PMID: 12007808

[ref27] Exss-Sonne P. Tolle J. Bader K. P. Pistorius E. K. Michel K. P. (2000). The IdiA protein of *Synechococcus* sp. PCC 7942 functions in protecting the acceptor side of photosystem II under oxidative stress. Photosynth. Res. 63, 145–157. doi: 10.1023/a:1006322925324, PMID: 16228425

[ref28] Falk S. Samson G. Bruce D. Huner N. P. Laudenbach D. E. (1995). Functional analysis of the iron-stress induced CP 43′ polypeptide of PS II in the cyanobacterium *Synechococcus* sp. PCC 7942. Photosynth. Res. 45, 51–60. doi: 10.1007/BF00032235, PMID: 24301379

[ref29] Feng X. Neupane B. Acharya K. Zazubovich V. Picorel R. Seibert M.. (2011). Spectroscopic study of the CP43′ complex and the PSI–CP43′ Supercomplex of the Cyanobacterium *Synechocystis* PCC 6803. J. Phys. Chem. B 115, 13339–13349. doi: 10.1021/jp206054b, PMID: 21978372

[ref30] Ferreira F. Straus N. A. (1994). Iron deprivation in cyanobacteria. J. Appl. Phycol. 6, 199–210. doi: 10.1007/BF02186073

[ref31] Foster J. S. Singh A. K. Rothschild L. J. Sherman L. A. (2007). Growth-phase dependent differential gene expression in *Synechocystis* sp. strain PCC 6803 and regulation by a group 2 sigma factor. Arch. Microbiol. 187, 265–279. doi: 10.1007/s00203-006-0193-6, PMID: 17160677

[ref32] Fournier G. P. Moore K. R. Rangel L. T. Payette J. G. Momper L. Bosak T. (2021). The Archean origin of oxygenic photosynthesis and extant cyanobacterial lineages. Proc. Biol. Sci. 288, 20210675. doi: 10.1098/rspb.2021.0675, PMID: 34583585 PMC8479356

[ref33] Fraser J. M. Tulk S. E. Jeans J. A. Campbell D. A. Bibby T. S. Cockshutt A. M. (2013). Photophysiological and photosynthetic complex changes during iron starvation in *Synechocystis* sp. PCC 6803 and *Synechococcus* elongatus PCC 7942. *PloS one* 8:11. doi: 10.1371/journal.pone.0059861 PMC3602374 23527279

[ref34] Ghassemian M. Straus N. A. (1996). Fur regulates the expression of iron-stress genes in the cyanobacterium *Synechococcus* sp. strain PCC 7942. Microbiology 142, 1469–1476. doi: 10.1099/13500872-142-6-1469 8704986

[ref35] González A. Fillat M. F. Bes M. -T. Peleato M. -L. Sevilla E. (2018). “The challenge of iron stress in cyanobacteria,” in Cyanobacteria. ed. Tiwari A.. 109–138.

[ref36] Guerinot M. L. Yi Y. (1994). Iron: nutritious, noxious, and not readily available. Plant Physiol. 104, 815–820. doi: 10.1104/pp.104.3.815, PMID: 12232127 PMC160677

[ref37] Guikema J. A. Sherman L. A. (1983). Organization and function of chlorophyll in membranes of cyanobacteria during iron starvation. Plant Physiol. 73, 250–256. doi: 10.1104/pp.73.2.250, PMID: 16663203 PMC1066448

[ref38] Guikema J. A. Sherman L. A. (1984). Influence of iron deprivation on the membrane-composition of anacystis-nidulans. Plant Physiol. 74, 90–95. doi: 10.1104/pp.74.1.90, PMID: 16663393 PMC1066630

[ref39] Guowei Q. Koedooder C. Qiu B.-S. Shaked Y. Keren N. (2021). Iron transport in cyanobacteria -from molecules to communities. Trends Microbiol. doi: 10.1016/j.tim.2021.06.001 34175176

[ref40] Hagemann M. Jeanjean R. Fulda S. Havaux M. Joset F. Erdmann N. (1999). Flavodoxin accumulation contributes to enhanced cyclic electron flow around photosystem I in salt-stressed cells of *Synechocystis* sp. strain PCC 6803. Physiol. Plant. 105, 670–678. doi: 10.1034/j.1399-3054.1999.105411.x

[ref41] Havaux M. Guedeney G. Hagemann M. Yeremenko N. Matthijs H. C. Jeanjean R. (2005). The chlorophyll-binding protein IsiA is inducible by high light and protects the cyanobacterium *Synechocystis* PCC6803 from photooxidative stress. FEBS Lett. 579, 2289–2293. doi: 10.1016/j.febslet.2005.03.021, PMID: 15848160

[ref42] Hernández J. A. Muro-Pastor A. M. Flores E. Bes M. T. Peleato M. L. Fillat M. F. (2006). Identification of a furA cis antisense RNA in the cyanobacterium *anabaena* sp. PCC 7120. J. Mol. Biol. 355, 325–334. doi: 10.1016/j.jmb.2005.10.079, PMID: 16324715

[ref43] Huner N. P. Krol M. Ivanov A. Sveshnikov D. Oquist G. (2001). CP43'induced under Fe-stress in *Synechococcus* sp. PCC 7942 is associated with PSI. *Science Access* 3, 3–61. doi: 10.1071/sa0403111

[ref44] Ihalainen J. A. D'Haene S. Yeremenko N. van Roon H. Arteni A. A. Boekema E. J.. (2005). Aggregates of the chlorophyll-binding protein IsiA (CP43') dissipate energy in cyanobacteria. Biochemistry 44, 10846–10853. doi: 10.1021/bi0510680, PMID: 16086587

[ref45] Ivanov A. Park Y.-I. Miskiewicz E. Raven J. Huner N. Öquist G. (2000). Iron stress restricts photosynthetic intersystem electron transport in *Synechococcus* sp. PCC 7942. FEBS Lett. 485, 173–177. doi: 10.1016/S0014-5793(00)02211-0, PMID: 11094162

[ref46] Ivanov A. G. Krol M. Selstam E. Sane P. V. Sveshnikov D. Park Y. I.. (2007). The induction of CP43' by iron-stress in *Synechococcus* sp. PCC 7942 is associated with carotenoid accumulation and enhanced fatty acid unsaturation. Biochimica et Biophysica Acta (BBA) 1767, 807–813. doi: 10.1016/j.bbabio.2007.02.006, PMID: 17362874

[ref47] Ivanov A. G. Krol M. Sveshnikov D. Selstam E. Sandström S. Koochek M.. (2006). Iron deficiency in cyanobacteria causes monomerization of photosystem I trimers and reduces the capacity for state transitions and the effective absorption cross section of photosystem I in vivo. Plant Physiol. 141, 1436–1445. doi: 10.1104/pp.106.082339, PMID: 16798943 PMC1533926

[ref48] Jantaro S. Ali Q. Lone S. He Q. (2006). Suppression of the lethality of high light to a quadruple HLI mutant by the inactivation of the regulatory protein PfsR in *Synechocystis* PCC 6803. J. Biol. Chem. 281, 30865–30874. doi: 10.1074/jbc.M606252200 16914546

[ref49] Jeanjean R. Zuther E. Yeremenko N. Havaux M. Matthijs H. C. Hagemann M. (2003). A photosystem 1 psaFJ-null mutant of the cyanobacterium *Synechocystis* PCC 6803 expresses the isiAB operon under iron replete conditions. FEBS Lett. 549, 52–56. doi: 10.1016/S0014-5793(03)00769-5, PMID: 12914924

[ref50] Jie W. Wei Z. Hui C. Jiao Z. He C. Wang Q. (2019). Ammonium nitrogen tolerant chlorella strain screening and its damaging effects on photosynthesis. Front. Microbiol. 9:03250. doi: 10.3389/fmicb.2018.03250 PMC6330332 30666245

[ref51] Jordan P. Fromme P. Witt H. T. Klukas O. Saenger W. Krauss N. (2001). Three-dimensional structure of cyanobacterial photosystem I at 2.5 A resolution. Nature 411, 909–917. doi: 10.1038/35082000, PMID: 11418848

[ref52] Karandashova I. Elanskaya I. Marin K. Vinnemeier J. Hagemann M. (2002). Identification of genes essential for growth at high salt concentrations using salt-sensitive mutants of the cyanobacterium *Synechocystis* sp. strain PCC 6803. Curr. Microbiol. 44, 184–188. doi: 10.1007/s00284-001-0035-3, PMID: 11821926

[ref53] Kojima K. Suzuki-Maenaka T. Kikuchi T. Nakamoto H. (2006). Roles of the cyanobacterial isiABC operon in protection from oxidative and heat stresses. Physiol. Plant. 128, 507–519. doi: 10.1111/j.1399-3054.2006.00781.x

[ref54] Kouril R. Arteni A. A. Lax J. Yeremenko N. D'Haene S. Rogner M.. (2005a). Structure and functional role of supercomplexes of IsiA and photosystem I in cyanobacterial photosynthesis. FEBS Lett. 579, 3253–3257. doi: 10.1016/j.febslet.2005.03.051, PMID: 15943969

[ref55] Kouril R. Yeremenko N. D'Haene S. Oostergetel G. T. Matthijs H. C. Dekker J. P.. (2005b). Supercomplexes of IsiA and photosystem I in a mutant lacking subunit PsaL. Biochimica et Biophysica Acta (BBA) 1706, 262–266. doi: 10.1016/j.bbabio.2004.11.008, PMID: 15694354

[ref56] Kouřil R. Yeremenko N. D'Haene S. Yakushevska A. E. Keegstra W. Matthijs H. C. P.. (2003). Photosystem I trimers from *Synechocystis* PCC 6803 lacking the PsaF and PsaJ subunits bind an IsiA ring of 17 units. Biochimica et Biophysica Acta (BBA) - Bioenergetics 1607, 1–4. doi: 10.1016/j.bbabio.2003.08.002, PMID: 14556907

[ref57] Krynicka V. Tichy M. Krafl J. Yu J. Kana R. Boehm M.. (2014). Two essential FtsH proteases control the level of the fur repressor during iron deficiency in the cyanobacterium *Synechocystis* sp. PCC 6803. Mol. Microbiol. 94, 609–624. doi: 10.1111/mmi.12782, PMID: 25238320

[ref58] Kuhn M. G. Vermaas W. F. J. (1993). Deletion mutations in a long hydrophilic loop in the photosystem-ii chlorophyll-binding protein CP43 in the cyanobacterium *Synechocystis* sp. PCC 6803. Plant Mol. Biol. 23, 123–133. doi: 10.1007/bf00021425, PMID: 8219045

[ref59] Kunert A. Vinnemeier J. Erdmann N. Hagemann M. (2003). Repression by fur is not the main mechanism controlling the iron-inducible isiAB operon in the cyanobacterium *Synechocystis* sp. PCC 6803. FEMS Microbiol. Lett. 227, 255–262. doi: 10.1016/S0378-1097(03)00689-X, PMID: 14592717

[ref60] Latifi A. Jeanjean R. Lemeille S. Havaux M. Zhang C.-C. (2005). Iron starvation leads to oxidative stress in *anabaena* sp. strain PCC 7120. J. Bacteriol. 187, 6596–6598. doi: 10.1128/JB.187.18.6596-6598.2005, PMID: 16159797 PMC1236655

[ref61] Laudenbach D. E. Straus N. A. (1988). Characterization of a cyanobacterial iron stress-induced gene similar to psbC. J. Bacteriol. 170, 5018–5026. doi: 10.1128/jb.170.11.5018-5026.1988, PMID: 3141374 PMC211566

[ref62] Lax J. E.-M. Arteni A. A. Boekema E. J. Pistorius E. K. Michel K.-P. Rögner M. (2007). Structural response of photosystem 2 to iron deficiency: characterization of a new photosystem 2–IdiA complex from the cyanobacterium *Thermosynechococcus elongatus* BP-1. Biochimica et Biophysica Acta (BBA)-Bioenergetics 1767, 528–534. doi: 10.1016/j.bbabio.2007.01.003, PMID: 17316552

[ref63] Legewie S. Dienst D. Wilde A. Herzel H. Axmann I. M. (2008). Small RNAs establish delays and temporal thresholds in gene expression. Biophys. J. 95, 3232–3238. doi: 10.1529/biophysj.108.133819, PMID: 18599624 PMC2547459

[ref64] Leonhardt K. Straus N. A. (1992). An iron stress operon involved in photosynthetic electron transport in the marine cyanobacterium *Synechococcus* sp. PCC 7002. J. Gen. Microbiol. 138, 1613–1621. doi: 10.1099/00221287-138-8-1613 1527503

[ref65] Leonhardt K. Straus N. A. (1994). Photosystem II genes isiA, psbDI and psbC in *anabaena* sp. PCC 7120: cloning, sequencing and the transcriptional regulation in iron-stressed and iron-repleted cells. Plant Mol. Biol. 24, 63–73. doi: 10.1007/BF00040574, PMID: 8111027

[ref66] Li Q. Huisman J. Bibby T. S. Jiao N. Z. (2019). Biogeography of Cyanobacterial isiA genes and their link to iron availability in the ocean. Front. Microbiol. 10. doi: 10.3389/fmicb.2019.00650 PMC6460047 31024472

[ref67] Lopez-Gomollon S. Hernandez J. A. Pellicer S. Angarica V. E. Peleato M. L. Fillat M. F. (2007). Cross-talk between iron and nitrogen regulatory networks in *anabaena* (Nostoc) sp. PCC 7120: identification of overlapping genes in FurA and NtcA regulons. J. Mol. Biol. 374, 267–281. doi: 10.1016/j.jmb.2007.09.010, PMID: 17920076

[ref68] Ma F. Zhang X. Zhu X. Li T. Zhan J. Chen H.. (2017). Dynamic changes of IsiA-containing complexes during long-term iron deficiency in *Synechocystis* sp. PCC 6803. Mol. Plant 10, 143–154. doi: 10.1016/j.molp.2016.10.009, PMID: 27777125

[ref69] MacGregor-Chatwin C. Jackson P. J. Sener M. Chidgey J. W. Hitchcock A. Qian P.. (2019). Membrane organization of photosystem I complexes in the most abundant phototroph on earth. Nature Plants 5, 879–889. doi: 10.1038/s41477-019-0475-z, PMID: 31332310 PMC6699766

[ref70] Martin-Luna B. Sevilla E. Gonzalez A. Bes M. T. Fillat M. F. Peleato M. L. (2011). Expression of fur and its antisense α-fur from Microcystis *aeruginosa* PCC7806 as response to light and oxidative stress. J. Plant Physiol. 168, 2244–2250. doi: 10.1016/j.jplph.2011.08.006, PMID: 21940066

[ref71] Melkozernov A. N. Bibby T. S. Lin S. Barber J. Blankenship R. E. (2003). Time-resolved absorption and emission show that the CP43'antenna ring of iron-stressed *Synechocystis* sp. PCC6803 is efficiently coupled to the photosystem I reaction center core. Biochemistry 42, 3893–3903. doi: 10.1021/bi026987u, PMID: 12667080

[ref72] Mills S. A. Marletta M. A. (2005). Metal binding characteristics and role of iron oxidation in the ferric uptake regulator from Escherichia coli. Biochemistry 44, 13553–13559. doi: 10.1021/bi0507579, PMID: 16216078

[ref73] Nealson K. H. Myers C. R. (1990). Iron reduction by bacteria - a potential role in the genesis of banded iron formations. Am. J. Sci. 290A, 35–45.

[ref74] Nield J. Morris E. P. Bibby T. S. Barber J. (2003). Structural analysis of the photosystem I supercomplex of cyanobacteria induced by iron deficiency. Biochemistry 42, 3180–3188. doi: 10.1021/bi026933k, PMID: 12641449

[ref75] Odom W. R. Hodges R. Chitnis P. R. Guikema J. A. (1993). Characterization of Synechocystis sp. PCC 6803 in iron-supplied and iron-deficient media. Plant Mol. Biol. 23, 1255–1264. doi: 10.1007/BF00042358, PMID: 8292789

[ref76] Pakrasi H. B. Goldenberg A. Sherman L. A. (1985a). Membrane development in the cyanobacterium, Anacystis nidulans, during recovery from iron starvation. Plant Physiol. 79, 290–295. doi: 10.1104/pp.79.1.290, PMID: 16664388 PMC1074868

[ref77] Pakrasi H. B. Riethman H. C. Sherman L. A. (1985b). Organization of pigment proteins in the photosystem II complex of the cyanobacterium Anacystis nidulans R2. Proc. Natl. Acad. Sci. 82, 6903–6907. doi: 10.1073/pnas.82.20.6903, PMID: 3931080 PMC390796

[ref78] Park Y. I. Sandström S. Gustafsson P. Öquist G. (1999). Expression of the isiA gene is essential for the survival of the cyanobacterium *Synechococcus* sp. PCC 7942 by protecting photosystem II from excess light under iron limitation. Mol. Microbiol. 32, 123–129. doi: 10.1046/j.1365-2958.1999.01332.x, PMID: 10216865

[ref79] Pilla S. K. Balaga R. R. Iwane S. Sisinthy S. Jogadhenu S. (2013). A novel transcriptional regulator, Sll1130, negatively regulates heat-responsive genes in *Synechocystis* sp. PCC6803. Biochem. J. 449, 751–760. doi: 10.1042/BJ20120928 23088579

[ref80] Rakhimberdieva M. G. Vavilin D. V. Vermaas W. F. Elanskaya I. V. Karapetyan N. V. (2007). Phycobilin/chlorophyll excitation equilibration upon carotenoid-induced non-photochemical fluorescence quenching in phycobilisomes of the cyanobacterium *Synechocystis* sp. PCC 6803. Biochimica et Biophysica Acta (BBA) 1767, 757–765. doi: 10.1016/j.bbabio.2006.12.007, PMID: 17240350

[ref81] Raven J. A. Evans M. C. Korb R. E. (1999). The role of trace metals in photosynthetic electron transport in O2-evolving organisms. Photosynth. Res. 60, 111–150. doi: 10.1023/A:1006282714942

[ref82] Riethman H. C. Sherman L. A. (1988). Immunological characterization of iron-regulated membrane proteins in the cyanobacterium Anacystis nidulans R2. Plant Physiol. 88, 497–505. doi: 10.1104/pp.88.2.497, PMID: 16666334 PMC1055607

[ref83] Ryan-Keogh T. J. Macey A. I. Cockshutt A. M. Moore C. M. Bibby T. S. (2012). The cyanobacterial chlorophyll-binding-protein IsiA acts to increase the in vivo effective absorption cross-section of PSI under iron limitation. J. Phycol. 48, 145–154. doi: 10.1111/j.1529-8817.2011.01092.x, PMID: 27009659

[ref84] Salomon E. Keren N. (2015). Acclimation to environmentally relevant Mn concentrations rescues a cyanobacterium from the detrimental effects of iron limitation. Environ. Microbiol. 17, 2090–2098. doi: 10.1111/1462-2920.12826, PMID: 25728137

[ref85] Sandström S. Ivanov A. G. Park Y. I. Öquist G. Gustafsson P. (2002). Iron stress responses in the cyanobacterium *Synechococcus* sp. PCC7942. Physiol. Plant. 116, 255–263. doi: 10.1034/j.1399-3054.2002.1160216.x, PMID: 12354203

[ref86] Sandström S. Park Y. I. Öquist G. Gustafsson P. (2001). CP43′, the isiA gene product, functions as an excitation energy Dissipator in the Cyanobacterium *Synechococcus* sp. PCC 7942. Photochem. Photobiol. 74, 431–437. doi: 10.1562/0031-8655(2001)074<0431:CTIGPF>2.0.CO;2, PMID: 11594057

[ref87] Sarcina M. Mullineaux C. W. (2004). Mobility of the IsiA chlorophyll-binding protein in cyanobacterial thylakoid membranes. J. Biol. Chem. 279, 36514–36518. doi: 10.1074/jbc.M405881200 15218021

[ref88] Schoffman H. Keren N. (2019). Function of the IsiA pigment-protein complex in vivo. Photosynth. Res. 141, 343–353. doi: 10.1007/s11120-019-00638-5, PMID: 30929163

[ref89] Schrader P. S. Milligan A. J. Behrenfeld M. J. (2011). Surplus photosynthetic antennae complexes underlie diagnostics of iron limitation in a cyanobacterium. PLoS One 6, e18753. doi: 10.1371/journal.pone.0018753, PMID: 21533084 PMC3080375

[ref90] Sevilla E. Martín-Luna B. González A. Gonzalo-Asensio J. A. Peleato M. L. Fillat M. F. (2011). Identification of three novel antisense RNAs in the fur locus from unicellular cyanobacteria. Microbiology 157, 3398–3404. doi: 10.1099/mic.0.048231-0 21921103

[ref91] Sherman D. Sherman L. (1983). Effect of iron deficiency and iron restoration on ultrastructure of Anacystis nidulans. J. Bacteriol. 156, 393–401. doi: 10.1128/jb.156.1.393-401.1983, PMID: 6413495 PMC215094

[ref92] Singh A. K. Li H. Bono L. Sherman L. A. (2005). Novel adaptive responses revealed by transcription profiling of a *Synechocystis* sp. PCC 6803 ΔisiA mutant in the presence and absence of hydrogen peroxide. Photosynth. Res. 84, 65–70. doi: 10.1007/s11120-004-6429-x, PMID: 16049756

[ref93] Singh A. K. Sherman L. A. (2006). Iron-independent dynamics of IsiA production during the transition to stationary phase in the cyanobacterium *Synechocystis* sp. PCC 6803. FEMS Microbiol. Lett. 256, 159–164. doi: 10.1111/j.1574-6968.2006.00114.x, PMID: 16487334

[ref94] Sun J. Golbeck J. H. (2015). The presence of the IsiA-PSI Supercomplex leads to enhanced photosystem I electron throughput in iron-starved cells of *Synechococcus* sp. PCC 7002. J. Phys. Chem. B 119, 13549–13559. doi: 10.1021/acs.jpcb.5b02176, PMID: 26046955

[ref95] Tölle J. Michel K.-P. Kruip J. Kahmann U. Preisfeld A. Pistorius E. K. (2002). Localization and function of the IdiA homologue Slr1295 in the cyanobacterium *Synechocystis* sp. strain PCC 6803. Microbiology 148, 3293–3305. doi: 10.1099/00221287-148-10-3293 12368463

[ref96] Tomitani A. (1999). Chlorophyll b and phycobilins in the common ancestor of cyanobacteria and chloroplasts. Nature 400, 159–162. doi: 10.1038/22101, PMID: 10408441

[ref97] Toporik H. Li J. Williams D. Chiu P.-L. Mazor Y. (2019). The structure of the stress-induced photosystem I-IsiA antenna supercomplex. Nat. Struct. Mol. Biol. 26, 443–449. doi: 10.1038/s41594-019-0228-8, PMID: 31133699

[ref98] Troyan T. Bullerjahn G. Sherman L. (1989). “Assembly of Chl-protein complexes in membranes of iron-stressed *Synechococcus* SP. PCC7942 proceeds in the absence of chlorophyll synthesis,” in Techniques and New Developments in Photosynthesis Research. (Springer), 601–604.

[ref99] Umena Y. Kawakami K. Shen J. R. Kamiya N. (2011). Crystal structure of oxygen-evolving photosystem II at a resolution of 1.9 A. Nature 473, 55–60. doi: 10.1038/nature09913, PMID: 21499260

[ref100] van der Weij-de Wit C. D. Ihalainen J. A. van de Vijver E. D’Haene S. Matthijs H. C. van Grondelle R.. (2007). Fluorescence quenching of IsiA in early stage of iron deficiency and at cryogenic temperatures. Biochimica et Biophysica Acta (BBA) 1767, 1393–1400. doi: 10.1016/j.bbabio.2007.10.001, PMID: 17980697

[ref101] Vik S. (2007). ATP synthesis by oxidative phosphorylation. EcoSal Plus 2, 177–231. doi: 10.1128/ecosalplus.3.2.3 26443583

[ref102] Vinnemeier J. Kunert A. Hagemann M. (1998). Transcriptional analysis of the isiAB operon in salt-stressed cells of the cyanobacterium *Synechocystis* sp. PCC 6803. FEMS Microbiol. Lett. 169, 323–330. doi: 10.1111/j.1574-6968.1998.tb13336.x, PMID: 9868777

[ref103] Wang Q. Hall C. L. Al-Adami M. Z. He Q. (2010). IsiA is required for the formation of photosystem I supercomplexes and for efficient state transition in synechocystis PCC 6803. PLoS One 5. doi: 10.1371/journal.pone.0010432 PMC2862709 20454661

[ref104] Wang Q. Jantaro S. Lu B. Majeed W. Bailey M. He Q. (2008). The high light-inducible polypeptides stabilize trimeric photosystem I complex under high light conditions in Synechocystis PCC 6803. Plant Physiol. 147, 1239–1250. doi: 10.1104/pp.108.121087, PMID: 18502976 PMC2442545

[ref105] Wang Q. Sun H. Huang J. L. (2017). Re-analyses of "algal" genes suggest a complex evolutionary history of Oomycetes. Front. Plant Sci. 8. doi: 10.3389/fpls.2017.01540 PMC5592239 28932232

[ref106] Wilson A. Ajlani G. Verbavatz J.-M. Vass I. Kerfeld C. A. Kirilovsky D. (2006). A soluble carotenoid protein involved in phycobilisome-related energy dissipation in cyanobacteria. The Plant Cell Online 18, 992–1007. doi: 10.1105/tpc.105.040121, PMID: 16531492 PMC1425857

[ref107] Wilson A. Boulay C. Wilde A. Kerfeld C. A. Kirilovsky D. (2007). Light-induced energy dissipation in iron-starved cyanobacteria: roles of OCP and IsiA proteins. The Plant Cell Online 19, 656–672. doi: 10.1105/tpc.106.045351, PMID: 17307930 PMC1867334

[ref108] Wrigglesworth J. M. Baum H. (1980). The biochemical functions of iron. Iron in Biochemistry and Medicine, 29–86.

[ref109] Xiao T. W. Mi M. M. Wang C. Y. Qian M. Chen Y. H. Zheng L. Q.. (2018). A methionine-R-sulfoxide reductase, OsMSRB5, is required for rice defense against copper toxicity. Environ. Exp. Bot. 153, 45–53. doi: 10.1016/j.envexpbot.2018.04.006

[ref110] Xu L. H. Wang W. Y. Guo J. J. Qin J. Shi D. Q. Li Y. L.. (2014a). Zinc improves salt tolerance by increasing reactive oxygen species scavenging and reducing Na+ accumulation in wheat seedlings. Biol. Plant. 58, 751–757. doi: 10.1007/s10535-014-0442-5

[ref111] Xu W.-L. Jeanjean R. Liu Y.-D. Zhang C.-C. (2003). pkn22 (alr2502) encoding a putative Ser/Thr kinase in the cyanobacterium *anabaena* sp. PCC 7120 is induced by both iron starvation and oxidative stress and regulates the expression of isiA. FEBS Lett. 553, 179–182. doi: 10.1016/s0014-5793(03)01019-6, PMID: 14550569

[ref112] Xu W. Chen H. He C.-L. Wang Q. (2014b). Deep sequencing-based identification of small regulatory RNAs in *Synechocystis* sp. PCC 6803. *PloS one*. 9:e92711. doi: 10.1371/journal.pone.0092711 PMC3960264 24647397

[ref113] Yeremenko N. Kouril R. Ihalainen J. A. D'Haene S. van Oosterwijk N. Andrizhiyevskaya E. G.. (2004). Supramolecular organization and dual function of the IsiA chlorophyll-binding protein in cyanobacteria. Biochemistry 43, 10308–10313. doi: 10.1021/bi048772l, PMID: 15301529

[ref114] Yousef N. Pistorius E. K. Michel K. P. (2003). Comparative analysis of idiA and isiA transcription under iron starvation and oxidative stress in *Synechococcus elongatus* PCC 7942 wild-type and selected mutants. Arch. Microbiol. 180, 471–483. doi: 10.1007/s00203-003-0618-4, PMID: 14605795

[ref115] Yu J. J. Zhang Y. X. Liu J. M. Wang L. Liu P. P. Yin Z. P.. (2018). Proteomic discovery of H2O2 response in roots and functional characterization of PutGLP gene from alkaligrass. Planta 248, 1079–1099. doi: 10.1007/s00425-018-2940-8, PMID: 30039231

[ref116] Zhang Y. Chen M. Church W. B. Lau K. W. Larkum A. W. D. Jermiin L. S. (2010). The molecular structure of the IsiA–photosystem I supercomplex, modelled from high-resolution, crystal structures of photosystem I and the CP43 protein. Biochimica et Biophysica Acta (BBA) - Bioenergetics 1797, 457–465. doi: 10.1016/j.bbabio.2010.01.002, PMID: 20064486

[ref117] Zhao L. S. Huokko T. Wilson S. Simpson D. M. Wang Q. Ruban A. V.. (2020). Structural variability, coordination and adaptation of a native photosynthetic machinery. Nature Plants 6, 869–882. doi: 10.1038/s41477-020-0694-3, PMID: 32665651

